# Histone peptide microarray screen of chromo and Tudor domains defines new histone lysine methylation interactions

**DOI:** 10.1186/s13072-017-0117-5

**Published:** 2017-03-14

**Authors:** Erin K. Shanle, Stephen A. Shinsky, Joseph B. Bridgers, Narkhyun Bae, Cari Sagum, Krzysztof Krajewski, Scott B. Rothbart, Mark T. Bedford, Brian D. Strahl

**Affiliations:** 1 Department of Biological and Environmental Sciences, Longwood University, Farmville, VA 23909 USA; 2 Department of Biochemistry and Biophysics, The University of North Carolina, Chapel Hill, NC 27599 USA; 3 Lineberger Comprehensive Cancer Center, The University of North Carolina School of Medicine, Chapel Hill, NC 27599 USA; 4 Department of Epigenetics and Molecular Carcinogenesis, The University of Texas MD Anderson Cancer Center, Smithville, TX 78957 USA; 5 Center for Epigenetics, Van Andel Research Institute, Grand Rapids, MI 49503 USA; 6 Roy & Diana Vagelos Laboratories, Department of Chemistry, University of Pennsylvania, Philadelphia, PA USA

**Keywords:** Chromatin, Histone methylation, Chromodomain, Tudor domain, Peptide microarray

## Abstract

**Background:**

Histone posttranslational modifications (PTMs) function to regulate chromatin structure and function in part through the recruitment of effector proteins that harbor specialized “reader” domains. Despite efforts to elucidate reader domain–PTM interactions, the influence of neighboring PTMs and the target specificity of many reader domains is still unclear. The aim of this study was to use a high-throughput histone peptide microarray platform to interrogate 83 known and putative histone reader domains from the chromo and Tudor domain families to identify their interactions and characterize the influence of neighboring PTMs on these interactions.

**Results:**

Nearly a quarter of the chromo and Tudor domains screened showed interactions with histone PTMs by peptide microarray, revealing known and several novel methyllysine interactions. Specifically, we found that the CBX/HP1 chromodomains that recognize H3K9me also recognize H3K23me2/3—a poorly understood histone PTM. We also observed that, in addition to their interaction with H3K4me3, Tudor domains of the Spindlin family also recognized H4K20me3—a previously uncharacterized interaction. Several Tudor domains also showed novel interactions with H3K4me as well.

**Conclusions:**

These results provide an important resource for the epigenetics and chromatin community on the interactions of many human chromo and Tudor domains. They also provide the basis for additional studies into the functional significance of the novel interactions that were discovered.

**Electronic supplementary material:**

The online version of this article (doi:10.1186/s13072-017-0117-5) contains supplementary material, which is available to authorized users.

## Background

Dynamic regulation of chromatin structure is linked to the regulation of all DNA-templated processes including gene expression [1, 2]. Histone posttranslational modifications (PTMs) represent one of the major mechanisms for regulating the chromatin landscape [3, 4]. Histone PTMs include acetylation, phosphorylation, and methylation among many others, and these different chemical signatures exert their effects on chromatin structure both in a type- and site-specific manner [5–8]. One of the primary mechanisms by which histone PTMs alter chromatin structure is via the recruitment of effector proteins that contain specialized “reader” domains that specifically recognize different histone PTMs [5, 9, 10]. These effectors may be transcription factors or additional chromatin-modifying machinery, and often their functions define the downstream consequences associated with distinct histone PTMs [10–12].

Histone lysine (K) methylation represents one of the major histone PTMs and over 170 methyllysine readers are thought to exist in humans [13]. Lysine methylation can take the form of mono-, di-, or trimethylation, and often each state is associated with distinct genomic locations and unique functional outcomes [14–16]. The major sites of K-methylation on histone H3 are K4, K9, K27, K36, and K79, while K20 is the predominant K-methylation site on histone H4 [14, 15, 17, 18]. Interestingly, histone K-methylation is associated with both transcriptionally permissive and transcriptionally repressive states of chromatin, dependent on the site and degree of methylation. For instance, monomethylation of histone H4 at Lys 20 (H4K20me1) is associated with actively transcribed regions, while trimethylation of H3K9 (H3K9me3) is associated with transcriptionally silent chromatin [19–23]. This suggests that methyllysine readers are specific both for the degree of methylation and for the sequence context surrounding the methylated residue. However, some sites of K-methylation, such as H3K9 and H3K27, share a common ARKS sequence context, suggesting that some reader domains may recognize multiple sites and may require additional factors for specificity. Indeed, many chromatin modifiers and transcriptional machinery contain multiple reader domains that simultaneously engage multiple histone PTMs in a form of “cross-talk” [24]. Furthermore, the residues flanking methyllysine hotspots are often subject to PTMs that may permit or impede binding of methyllysine readers. For example, phosphorylation of serine 10 of H3 (H3S10p) inhibits binding of H3K9me3-specific reader domains, and cis-histone tail H3K9me3/S10p has been observed in cells [25, 26]. Similarly, phosphorylation of H3T3, and to a lesser degree H3T6, impedes binding of H3K4me3-specific reader domains, while symmetrical or asymmetrical dimethylation of H3R2 (H3R2me2s/a) has little effect on certain domain interactions [27], but can block or promote others [28–30].

Known methyllysine reader domains include the plant homeodomain (PHD) fingers, the bromo-adjacent homology (BAH) domains, and the “Royal family” domains [17, 31, 32]. The Royal family of methyllysine readers is conserved throughout eukaryotic evolution and includes the chromo-, Tudor-, PWWP-, and malignant brain tumor (MBT)-structural domains [33]. Members of this family are known to interact with multiple different sites of K-methylation on histones and other proteins. Structural and mechanistic studies of the heterochromatin protein 1 (HP1) chromodomain provided some of the first insights into the molecular mechanism of methyllysine recognition. A co-crystal structure of the HP1 chromodomain bound to an H3K9me3 peptide revealed that a hydrophobic region composed of three conserved aromatic residues stabilizes the interaction with the methyllysine side chain via cation–π and hydrophobic interactions [34]. Additional structure–function studies showed that this “aromatic cage” is a general feature of many methyllysine readers even outside the Royal family [35]. However, the site- and methyl-state specificity for many members of the Royal family is unclear. Moreover, several of the known members of this family are uncharacterized for their interactions with histone PTMs.

While multiple histone K-methylation sites are known, advances in mass spectrometry have increased the number of known sites [36]. However, it is unclear whether the current repertoire of known methyllysine readers, including members of the Royal family, also interacts with these newly identified sites or whether there are distinct readers for each site. In this investigation, we set out to use a high-throughput proteomics approach utilizing customized histone peptide microarrays to survey a large number of human chromo and Tudor domains for their binding to various histone PTMs. We identified several new histone PTM–reader domain interactions for previously characterized readers and identified a subset of H3K9-methyl readers that are also capable of reading H3K23 methylation—a newly identified yet poorly understood histone PTM. The results of our survey will facilitate future studies characterizing the structure–function relationships and biological consequences of these interactions.

## Results

We selected 31 chromodomains and 39 Tudor or Tudor-like domains to screen using our histone peptide microarray platform. Several additional protein reader domains were also screened, for a total of 83 proteins screened by peptide microarray (Additional file 1: Table S1). Our peptide microarray platform consists of nearly 300 biotinylated histone peptides harboring unique PTMs at one or more residues immobilized to a streptavidin-coated glass surface (Additional file 2: Table S2) [37, 38]. The arrays were probed in duplicate with purified GST-tagged protein domains, and binding to specific PTMs was detected using fluorescently labeled antibodies (see Methods). Twenty-two of the 83 selected domains showed positive hits with one or more histone peptides on the arrays. As shown in Table 1, many of the known binding targets for these protein domains were detected, as well as several novel interactions. Based on these observations, we focused on further characterizing (1) the novel interaction between Spindlin1 (SPIN1) triple Tudor domain and H4K20me3 and (2) the interaction between several chromodomains and H3K23me2/3.

**Table 1:** Summary of reader domain interactions identified via histone peptide microarrays

Protein	Domain	Top array hits	Known interactions	References
HP1β/CBX1	Chromo	H3K9me1/2/3, H3K23me1/2/3	H3K9me1/2/3, H3K23me1/2/3	[21, 22, 46, 52]
HP1γ/CBX3	Chromo	*H3K9me1/2/3*, *H3K23me3* ^a^	H3K9me2/3	[21, 22, 52, 74]
HP1α/CBX5	Chromo	*H3K9me1/2/3*, *H3K23me3* ^a^	H3K9me1/2/3	[21, 48, 52]
CDYL1b	Chromo	*H3K9me1/2/3*, *H3K27me2/3*	H3K9me1/2/3, H3K27me1/2/3	[75]
CDYL2	Chromo	*H3K9me2/3*, *H3K27me3*	H3K9me1/2/3, H3K27me1/2/3	[76]
CHD1	Chromo (2)	H3K4me2/3	H3K4me3	[54, 55]
CHD7	Chromo (2)	H4me0	H3K4me1/2/3	[56]
CHD9	Chromo (2)	H4me0		
MPP8	Chromo	*H3K9me1/2/3*, *H3K23me2/3*	H3K9me1/2/3, H3K23me1/2/3	[46, 77, 78]
53BP1	Tudor (2)	*H3K4me2*, H3K18me2^a^, H3K36me2^a^, *H4K20me1/2*	H3K4me2, H4K20me1/2	[42, 52]
JMJD2A	Tudor (2)	*H3K4me2/3*, H3K18me3^a^, H3K9me3, *H4K20me2/3*	H3K4me3, H4K20me2/3	[51, 52]
PHF20	Tudor (2)	H3K9me2/3, H4K8me1	H3K9me2, H3K27me2, H3K36me2	[35]
UHRF1	Tudor-like	H3K9me2/3	H3K9me2/3	[79, 80]
PHF1	Tudor	H3K36me3	H3K36me2/3	[81–84]
SGF29	Double Tudor	*H3K4me3*	H3K4me1/2/3	[85]
SPIN1	Triple Tudor	*H3K4me2/3*, H3K18me3, *H4K20me3* ^a^	H3K4me2/3	[40]
TDRD2	Tudor (extended)	H3K4me3		
TDRD3	Tudor	H3R2me2a, H3R8me2a	pan-Rme2a	[53]
GLP	ANK	H3K9me1/2	H3K9me1/2	[86]
ING2	PHD	H3K4me2/3	H3K4me2/3	[87, 88]
TAF3	PHD	*H3K4me2/3*	H3K4me2/3	[89]
L3MBTL1	MBT (3)	H3K4me2, H3K9me2, H4K8me1	H3K4me1/2, H3K9me1/2, H3K27me1/2, H4K20me1/2	[90–92]

Italics, validated by peptide pull-down assays

^a^Novel interactions

**Fig. 1:**
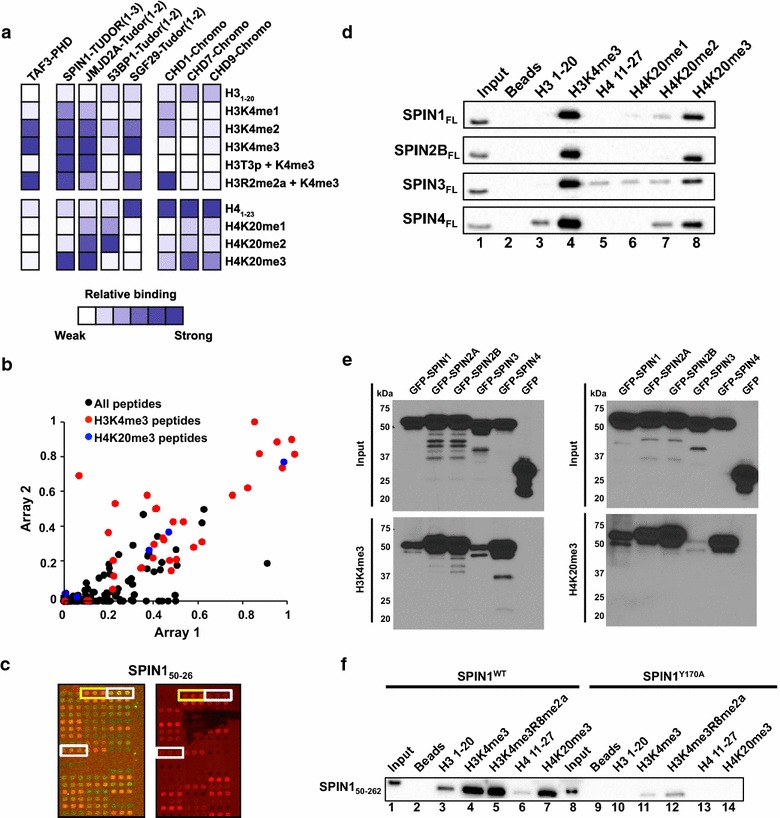
SPIN1 triple Tudor domain interacts with H4K20me3 as well as H3K4me3. **a** Heat map showing the relative binding detected for each of the indicated domains on the peptide microarray platform. Data represent the average of two independent arrays relative to the most intense binding signal within the indicated set of peptides. **b** Scatter plot of the relative binding of SPIN1 triple Tudor domain from two independent peptide arrays. H3K4me3-containing peptides are shown in red and H4K20me3-containing peptides are shown in *blue*. All other peptides are shown in *black*. **c** Representative picture of a section of the peptide microarray for SPIN1 triple Tudor domain. The *left panel* shows both the *green* (peptide) and *red* (protein binding) fluorescent channels, while the *right panel* depicts only the red fluorescence channel for clarity. Positive antibody controls are outlined in *white* and the positive interaction with the H4K20me3 peptide is outlined in *yellow*. Full array images are shown in Additional file 3: Figure S1.** d** Western blot results of peptide pull-down experiments with purified full-length Spindlin family members. The input is shown in *Lane 1* and the corresponding bound fraction is shown in *Lanes 2*–*8*. **e** Western blot results of peptide pull-down experiments with whole cell lysates derived from transiently transfected HEK 293T cells (GFP-SPIN1, 2A, 2B, 3 and 4). **f** Western blot results of peptide pull-down experiments with purified SPIN1 wild type (SPIN1^WT^) or aromatic cage mutant in the second Tudor domain (SPIN1^Y170A^) demonstrating a loss of H3K4me3 and H4K20me3 in the mutant

SPIN1 is a transcriptional coactivator that contains a triple Tudor domain known to interact with H3K4me3—a mark associated with actively transcribed genes [39–41]. As expected, the SPIN1 triple Tudor and several other known H3K4me-binding domains, including the PHD domain of Taf3 and the tandem Tudor domains of JMJD2A and SGF29, strongly interacted with H3K4me2/3 (Fig. 1a; Additional file 3: Figure S1, and Additional file 4: Figure S2). Interestingly, phosphorylation of H3T3 inhibited binding of SGF29 tandem Tudor and Taf3 PHD, but did not interfere with SPIN1 triple Tudor or JMJD2A tandem Tudor interaction with H3K4me3. In contrast, all of the H3K4me3 reader domains, including CHD1 chromodomain, accommodated H3K4me3 with neighboring H3R2me2a. Furthermore, the chromodomains of CHD7 and CHD9 showed robust interaction with both the unmodified H3 and H4N-terminal tails, while the chromodomain of CHD1 showed interaction with the H4 tail but not with the unmodified H3 tail (Fig. 1a; Additional file 5: Figure S3, Additional file 6: Figure S4, and Additional file 7: Figure S5). In addition, the tandem Tudor of 53BP1 preferentially bound H4K20me1/2 as previously described (Fig. 1a) [42].

Surprisingly, the SPIN1 triple Tudor domain also bound H4K20me3-containing peptides (Fig. 1b, c). This novel interaction was highly reproducible on the peptide microarrays and was confirmed by in-solution peptide pull-down assays (Additional file 3: Figure S1A and S1B). We further validated the specificity of the interaction using peptide pull-down experiments with full-length SPIN1 protein and tested the possibility that other Spindlin family members (SPIN2B, SPIN3, and SPIN4) interact with H4K20me3. Indeed, full-length SPIN1 protein preferentially bound H4K20me3 and showed less interaction with mono- or dimethylated H4K20 (Fig. 1d). Like SPIN1, full-length SPIN2B, SPIN3, and SPIN4 also showed preference for trimethylated H3K4 and H4K20 (Fig. 1d). In order to test whether SPIN1 family members interact with H4K20me3 peptides in the context of the cellular milieu, we transfected 293T cells with constructs containing individual SPIN1 family proteins fused with green fluorescent protein (GFP) and performed peptide pull-down experiments with whole cell lysates. The results from these assays mimic the results obtained using purified proteins, further suggesting that SPIN1 family proteins preferentially interact with H3K4me3 and H4K20me3 in cells (Fig. 1e; Additional file 3: Figure S1C). Notably, while we were unable to purify SPIN2A to high enough quality for use in in vitro pull-down assays using purified proteins, we were able to assess SPIN2A binding to H3K4me3/H4K20me3 peptides using lysates from transfected cells. These results show that, like other SPIN1 family members, SPIN2A is capable of interacting with both H3K4me3 and H4K20me3 (Fig. 1e).

Previous crystallographic analysis of the interaction between SPIN1 triple Tudor and H3K4me3 revealed an aromatic cage in the second Tudor domain of the SPIN1 triple Tudor that coordinates the trimethylated lysine residue [41]. In order to determine whether the interaction with H4K20me3 occurs through the same aromatic cage, we generated a tyrosine 170 to alanine (Y170A) mutation in the aromatic cage of SPIN1 and tested the interaction with H4K20me3 using peptide pull-down analyses. As shown in Fig. 1f, the Y170A variant showed minimal interaction with both H3K4me3 and H4K20me3. The SPIN1 Y170A mutation was previously shown to disrupt H3K4me3 binding [43]; thus, our results suggest that H4K20me3 is coordinated in the same aromatic pocket. Additional structural and functional studies show that H3R8me2a enhances the binding of SPIN1 to H3K4me3-containing peptides [43]. The methylated Arg8 side chain is coordinated in a hydrophobic pocket in the first Tudor domain of SPIN1, while the H3K4me3 residue is coordinated in the second Tudor domain [43]. In order to confirm that the Y170A mutant specifically disrupts the second Tudor domain, we tested the interaction of SPIN1 with a peptide containing the double H3K4me3/H3R8me2a modification. Both wild-type SPIN1 and the Y170A variant showed enhanced interaction with the H3K4me3/H3R8me2a peptide compared to the H3K4me3 peptide, suggesting specific disruption of the second Tudor domain in the Y170A variant (Fig. 1f). Together, these results indicate that the second Tudor domain of SPIN1 can bind both H3K4me3 and H4K20me3 in a mutually exclusive manner.

**Fig. 2:**
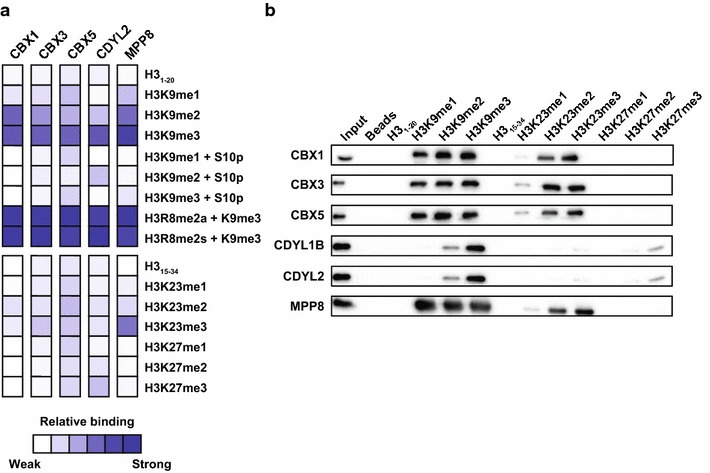
Chromodomains interact with H3K23me2/3 in addition to H3K9me1/2/3. **a** Heat map showing the relative binding detected for each of the indicated domains on the peptide microarray platform. Data represent the average of two independent arrays relative to the most intense binding signal within the indicated set of peptides. **b** Western blot results of peptide pull-downs performed with the indicated GST-tagged domain and histone peptide. The input is shown in *Lane 1* and the bound fraction is shown in *Lanes 2*–*13*

The second novel interaction we detected occurred between several chromodomains and H3K23me peptides. As shown in Fig. 2a, the chromodomains of CBX1, CBX3, CBX5, CDYL2, and MPP8 showed strong interaction with H3K9me3, even in the context of neighboring arginine 8 asymmetrical or symmetrical dimethylation. In all cases, H3S10phos inhibited the interaction with H3K9me3 and there was a clear preference for H3K9me3 over H3K27me3. In addition to the well-characterized interactions with H3K9me3, we also observed binding to H3K23me-containing peptides. To validate these interactions, we performed peptide pull-down experiments with H3K9, H3K23, and K3K27 peptides with varied degrees of methylation. As shown in Fig. 2b, we observed that CBX1, CBX3, CBX5, and MPP8 chromodomains interacted with H3K9me1/2/3 peptides and H3K23me2/3 peptides, but showed minimal interactions with H3K27me1/2/3 peptides. CDYL1B and CDYL2 showed preference for H3K9me2/3 peptides and very weak interactions with H3K23me peptides and H3K27me peptides. These results suggest that members of the CBX family of H3K9me3 reader domains, which have high selectivity for H3K9 over a very similar sequence motif at H3K27 (i.e., ARKS), are robust readers of H3K23 methylation, thus implicating H3K23 methylation, along with H3K9me, in the silencing functions of these domains.

## Discussion

The aim of this study was to create a valuable resource of chromo and Tudor reader domains for their interactions and cross-talk between histone PTMs. This work was facilitated by the use of a high-throughput approach employing peptide microarrays containing nearly 300 biotinylated histone peptides harboring up to five PTMs on each peptide (Additional file 2: Table S2). While several other histone peptide microarray platforms have been described [44–47], there are several notable features of our peptide array platform that aided the current study. These include highly purified peptides of lengths greater than 20 amino acids, along with each peptide being spotted multiple times by multiple pins to provide a robust number of data points that gave us high confidence in the interactions (and changes in these interactions by neighboring PTMs) that we observed.

Our survey of histone reader domains is one of the largest screens for histone PTM–reader domain interactions to date. We expressed and purified 83 protein domains, including 31 chromodomains and 39 Tudor or Tudor-like domains. We screened each domain in duplicate, and 22 domains exhibited consistent, reproducible binding to histone peptides on our arrays. The majority of the protein domains we tested, however, did not exhibit binding to histone peptides (see full list of domains screened in Additional file 1: Table S1). There are several possible explanations for this. First, our previous observations suggest that binding affinities weaker than approximately 30 µM are typically beyond the limit of detection for this platform [37]. It is notable that many reader domains exhibit weak interactions with histone peptides, which may account for a substantial number of negatives in our screen. For example, the chromodomain of CBX2 has been shown to bind H3K9me3 and H3K27me2 peptides with a binding affinity of ~40 µM via fluorescence polarization [48], which would explain why this chromodomain failed to show PTM interactions as compared to the other CBX domains. Second, we screened several protein domains with unknown histone PTM binding targets. For example, the Tudor domains of TDRD1 and TDRD2 are known to interact with methylated Piwi proteins [49, 50], but there are no known methyl-histone binding targets known to date. Similarly, TDRD4, TDRD9, and several other TDRD family members have no known methyl-histone binding targets, and it is possible that these Tudor domains do not interact with histones. Third, the recombinant protein domains we expressed and purified may require additional sequences from their respective proteins that are needed for histone PTM binding and are not present in the domains we designed. Indeed, the single Tudor domain of PHF20 was negative on our arrays, but the tandem Tudor domain interacted with H3K9me2, as previously shown [35]. In addition, the domains we purified may require interaction with other proteins in order to bind histones. Finally, it is possible that the conditions we used in this high-throughput approach were not amenable to binding for some proteins.

Of the 31 Tudor or Tudor-like domains we screened, several known interactions were detected on our arrays (Table 1). Both 53BP1 and JMJD2A tandem Tudor domains showed binding to H3K4me and H4K20me peptides as previously shown [42, 51, 52]. Some novel interactions were also detected on the peptide arrays, such as binding to H3K18me, but further experiments need to be performed to validate these findings. TDRD3 Tudor domain specifically recognized asymmetrically dimethylated peptides, as previously shown [53], but our results suggest that this Tudor domain has broad affinity for Rme2a-containing peptide (Additional file 8: Figure S6 and Table 1). Of the 39 chromodomains we surveyed, nearly 20% interacted with modified histone peptides. Many of these interactions are well characterized, such as binding to H3K9me peptides by the CBX family of proteins [21, 22, 52]. We also observed interaction between CHD1 chromodomain and H3K4me3 as previously described [54, 55]. Intriguingly, the chromodomains of CHD1, CHD7, and CHD9 all showed interactions with unmodified histone H4 peptide, and CHD7 and CHD9 also interacted with unmodified histone H3 peptide (Additional file 5: Figure S3, Additional file 6: Figure S4, Additional file 5: Figure S5, and Table 1). Although CHD9 chromodomains have not been shown to bind methylated histones, H3K4me1/2/3 peptides were shown to competitively disrupt histone interactions with purified histones, which is in agreement with the idea that the H3N terminus can bind the chromodomain of CHD9 [56]. It should be noted that unmodified histone H3 peptides were not tested in these experiments, but based on our findings, we speculate the K4 unmodified peptide would have also competed CHD9 chromodomain interaction given our results show general H3 binding without preference to the H3K4 methyl state.

Due to the large scope of this microarray screen, we focused on validating only a subset of interactions by peptide pull-down experiments. The two most significant and novel interactions we uncovered were with H3K23me and H4K20me peptides. First, we observed that several chromodomains interacted with H3K23me, in addition to the known H3K9me targets. MPP8 and CBX1 chromodomains have been shown to interact with H3K23me peptides at low micromolar binding affinities [46]. We confirmed these results in our screen and observed that additional chromodomains, CBX3 and CBX5, also interact with H3K23me2/3. In our study, we observed preferential binding to di- and trimethylated states of H3K23. All three methylation states of H3K23 have been detected in cells [46], and a recent report suggests that H3K23me3 colocalizes with H3K27me3 and plays a role in protecting heterochromatin from double-strand DNA breaks during meiosis [57]. It is possible that the dual modification may provide an even better binding substrate for these chromodomains, which will be interesting to explore in future biological studies. In contrast, the CDY family members that exhibited binding on our arrays (CDYL1B and CDYL2) showed preference for H3K9me3 and did not interact with H3K23me peptides. Notably, a recent report suggested that H3K23 methylation regulates levels of H3K36 methylation by recruiting the H3K36 demethylase, KDM4B, via its double Tudor domain [58]. However, none of the Tudor domains surveyed here showed interaction with H3K23 methylation.

We also focused on the novel interaction between the SPIN1 Tandem Tudor domain and H4K20me2/3, which we validated by peptide pull-down experiments and demonstrated with other Spindlin family members (Fig. 1d). In the context of full-length protein, SPIN1 shows remarkable preference for trimethylation at H3K4 and H4K20. SPIN2B and SPIN3 show similar selectivity, while SPIN4 seems to accommodate both di- and trimethylated H4K20me3. SPIN1 is composed of three homologous Tudor domains denoted I, II, and III [39]. The second Tudor domain (II) is composed of the aromatic residues Phe 141, Trp 151, Tyr 170, and Tyr 177, which together form the aromatic cage that coordinates the methyl lysine [41]. Our observation that the Tyr170Ala variant loses interaction with both H3K4me3 and H4K20me3 suggests that domain II is responsible for recognizing both modifications. This further suggests that SPIN1 interacts with H3K4me3 and H4K20me3 at different times and/or different genomic locations, possibly under different cellular conditions.

Lysine 20 is the predominant methylation site on H4, and this modification is important for development in higher eukaryotes (reviewed in [59]). H4K20 methylation is associated with regulating transcription, the DNA damage response, and cell cycle progression [59]. Multiple H4K20 methyl readers have been described, and many of them contain a Tudor or Tandem Tudor domain like SPIN1 [59]. These readers of H4K20me are thought to mediate the cellular roles ascribed to H4K20 methylation. For instance, the Tandem Tudor domain of 53BP1 is required for localization of 53BP1 to sites of DNA damage where it acts as a mediator of DNA damage signaling and repair [42, 60, 61]. Interestingly, different levels of H4K20 methylation are associated with different effects on transcription. For instance, H4K20me1 is associated with active transcriptional states of chromatin, while H4K20me3 is associated with transcriptionally silent chromatin regions [19, 62, 63]. Our data suggest that SPIN1 and other Spindlin family members are capable of recognizing both a transcriptional activation modification (i.e., H3K4me3) and a transcriptional silencing modification (i.e., H4K20me3). This result is, to the best of our knowledge, the first example of a reader domain that can read both activating and deactivating histone PTMs.

As H4K20 methylation is important in several cellular processes, aberrant H4K20 methylation is observed in several cancers and mutations in H4K20 methyl reader domains have been described in human developmental disorders [64–66]. Furthermore, SPIN1 is overexpressed in several varieties of malignant tumors and upregulation of SPIN1 is known to increase cellular proliferation and cause chromosomal instability and abnormal mitosis [67–71]. The interaction of SPIN1 with H4K20me2/3 may play a part in mediating some of the roles associated with H4K20 methylation, but additional in vivo studies are needed to determine the biological importance of this interaction. Chemical probes that inhibit the interaction of SPIN1 with H3K4me3 peptides in vitro have been described [72, 73]. Our work suggests that these probes could also be useful tools for characterizing the SPIN1-H4K20me interaction in vivo.

## Conclusions

This high-throughput screen aimed to determine the histone PTM binding targets of known and putative chromo and Tudor reader domains to create a valuable resource for future studies of these domains. Our survey encompassed the majority of human chromo and Tudor domains, and uncovered known and unknown histone PTM interactions. Of the many hits we observed, we focused on two novel interactions: (1) chromodomain recognition of H3K23me and (2) recognition of H4K20me3 by the Spindlin family of proteins. Future work will be needed to uncover the importance of these interactions in vivo.

## Methods

### Protein expression and purification

Codon optimized constructs were synthesized and cloned into pGEX-4T-1 expression vectors (GE Healthcare) by Biomatik. Proteins were expressed in soluBL21 (DE3) (Amsbio) grown in Terrific Broth II media (MP Biomedicals). After culturing at 37 °C until an OD_600_ of ~0.6, cells were chilled for 30 min at 4 °C before induction with 1 mM IPTG for 20 h at 16 °C. Cells were harvested by centrifugation and pellets were flash frozen in liquid nitrogen. For purification, thawed cell pellets were resuspended in binding buffer (50 mM Tris pH 7.5, 250 mM NaCl, 4 mM DTT, 10% glycerol) supplemented with a protease inhibitor cocktail (Roche), 0.1 mM phenylmethane sulfonyl fluoride (PMSF), 0.5 mg/ml chicken egg lysozyme (Sigma), and 0.2% (v/v) Triton X-100. After incubation on ice for 45 min, cells were lysed by sonication and clarified by centrifugation. Lysates were incubated with glutathione agarose (Pierce) and then washed with 10 bed volumes of binding buffer. Bound protein was eluted with elution buffer (50 mM Tris pH 8.0, 250 mM NaCl, 4 mM DTT, 10% glycerol, 10 mM reduced glutathione) and then dialyzed against 3 l of binding buffer at 4 °C. Samples were concentrated by centrifugation and protein concentration and purity were determined by Bradford Assay (BioRad) and SDS-PAGE, respectively.

### Peptide microarrays

The peptide microarrays were generated and assayed as described previously [37, 38], except that the arrays contained four triplicate spots of each peptide. Briefly, GST-tagged proteins were diluted to 0.5–2 μM in phosphate-buffered saline (PBS) supplemented with 0.1% (v/v) Tween-20 (PBST) and 5% (w/v) bovine serum albumin (BSA, EMD Millipore Omnipure Fraction V) and incubated with peptide microarrays overnight at 4 °C. Arrays were washed three times with PBS and then probed with an anti-GST antibody (EpiCypher Inc.; Cat. No. 13-0022) diluted to 1:1000 in PBST + 5% BSA. Arrays were washed again 3× with PBS and then probed with an Alexa Fluor 647-conjugated anti-rabbit antibody at 1:10,000 (ThermoFisher). Arrays were imaged using a Typhoon Scanner and protein binding was determined as previously described [37, 38]. The average signal intensity for each peptide was normalized to the most intense binding within an array, and normalized binding was averaged for at least two independent replicates for each protein. Heat maps of relative binding were generated using JavaTree View (version 1.16r4) after normalizing the relative binding within the subset of peptides selected for the heat map.

### In-solution peptide pull-down assays

For pull-down experiments using purified proteins, a total of 50 pmol of GST-tagged protein was incubated with 500 pmols of biotinylated histone peptide for 1 h at 4 °C in peptide binding buffer (50 mM Tris pH 8.0, 300 mM NaCl, 0.1% NP-40). Following incubation, the protein–peptide mixture was incubated with streptavidin-coated magnetic beads (Pierce) and pre-equilibrated with peptide binding buffer, for 1 additional hour at 4 °C. The beads were washed three times with peptide binding buffer, and bound complexes were eluted with 1x SDS loading buffer, then resolved via SDS-PAGE and transferred to a PVDF membrane. The membrane was probed with an anti-GST antibody (EpiCypher Inc.; Cat. No. 13-0022) diluted 1:4000 in PBST supplemented with 5% (w/v) BSA (Sigma).

For pull-down experiments using cell lysates, HEK 293T cells were transiently transfected with GFP-SPIN1, 2A, 2B, 3, and 4 using polyethylenimine according to manufacturer’s instructions. Cells were lysed in ice-cold mild lysis buffer (50 mM Tris HCl pH 7.5, 150 mM NaCl, 0.1% NP-40, 5 mM EDTA, 5 mM EGTA, 15 mM MgCl2) containing protease inhibitor cocktail (Roche). Thirty microliter of streptavidin agarose beads (Millipore) was pre-washed with binding buffer and incubated with 10 µg of biotinylated histone peptides for 2 h with rocking at 4 °C. The beads were then washed three times with 500 μl binding buffer to remove unbound peptide. The peptide–streptavidin agarose mix was then incubated overnight with the whole cell lysates and rocked at 4 °C. After three washes with 500 μl binding buffer, 30 μl of 2× SDS loading buffer was added to the beads and boiled. The samples were subjected to SDS-PAGE and Western blotting analysis using polyclonal GFP antibody (Santa Cruz Biotech, 1:3000).

## Additional files

**Additional file 1: Table S1.** Protein domains screened by histone peptide microarray.

**Additional file 2: Table S2.** Histone peptide library used for peptide microarrays.

**Additional file 3: Figure S1.** SPIN1 Tudor domain interaction with H3K4me3 and H4K20me3. A) Representative array images of SPIN1 Tudor domain showing peptide binding indicated in red (right panel). The peptide tracer is shown in green (left panel). Positive antibody controls are outlined in white. B) Western blot results of peptide pull-down experiments with SPIN1 tandem Tudor domain. The input is shown in Lane 1 and the corresponding bound fraction is shown in Lanes 2–8. C) Western blot results of peptide pull-down experiments with whole cell lysates derived from transiently transfected HEK 293T cells (GFP-SPIN1, 2A, 2B, 3 and 4).

**Additional file 4: Figure S2.** Peptide microarray data for SGF29 and Taf3. Representative array images of A) SGF29 double Tudor domain and Taf3 PHD domain showing peptide binding indicated in red (right panel). The peptide tracer is shown in green (left panel). Positive antibody controls are outlined in white.

**Additional file 5: Figure S3.** CHD1 chromodomain histone peptide microarray. A) Representative array images of CHD1 chromodomain showing peptide binding indicated in red (right panel). The peptide tracer is shown in green (left panel). Positive antibody controls are outlined in white. B) Scatter plot of the relative binding of CHD1 chromodomain from two independent peptide arrays. All modified and unmodified H4 (1–23) peptides are shown in blue, and H3K4me2/3-containing peptides are shown in red. All other peptides are shown in black. C) Relative binding to the indicated histone peptides from two representative arrays. Data were normalized to the most intense binding and the average and standard deviation of triplicate spots is shown.

**Additional file 6: Figure S4.** CHD7 chromodomain histone peptide microarray. A) Representative array images of CHD7 chromodomain showing peptide binding indicated in red (right panel). The peptide tracer is shown in green (left panel). Positive antibody controls are outlined in white. B) Scatter plot of the relative binding of CHD7 chromodomain from two independent peptide arrays. All modified and unmodified H4 (1–23) peptides are shown in red. All other peptides are shown in black. C) Relative binding to the indicated histone peptides from one representative array. Data were normalized to the most intense binding and the average and standard deviation of triplicate spots is shown.

**Additional file 7: Figure S5.** CHD9 chromodomain histone peptide microarray. A) Representative array images of CHD9 chromodomain showing peptide binding indicated in red (right panel). The peptide tracer is shown in green (left panel). Positive antibody controls are outlined in white. B) Scatter plot of the relative binding of CHD9 chromodomain from two independent peptide arrays. All modified and unmodified H4 (1–23) peptides are shown in red. All other peptides are shown in black. C) Relative binding to the indicated histone peptides from one representative array. Data were normalized to the most intense binding and the average and standard deviation of triplicate spots is shown.

**Additional file 8: Figure S6.** TDRD3 Tudor domain histone peptide microarray. A) Representative array images of TDRD3 Tudor domain showing peptide binding indicated in red (right panel). The peptide tracer is shown in green (left panel).

## Abbreviations

BAH: bromo-adjacent homology
GST: glutathione *S*-transferase
MBT: malignant brain tumor
PBS: phosphate-buffered saline
PHD: plant homeodomain
PTM: posttranslational modification
