# Supplementary figures and images for: Histone peptide microarray screen of chromo and Tudor domains defines new histone lysine methylation interactions
Source: Epigenetics Chromatin. 2017 Mar 14;10:12. doi: 10.1186/s13072-017-0117-5 (PMC5348760; doi:10.1186/s13072-017-0117-5)

# Figure S1

**A**

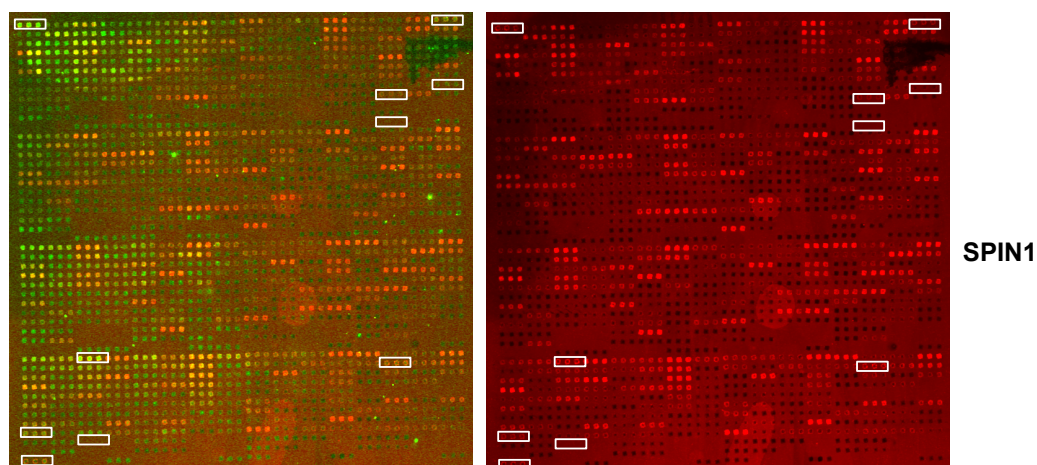

**B**

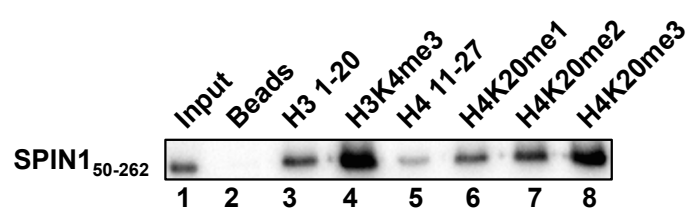

**C**

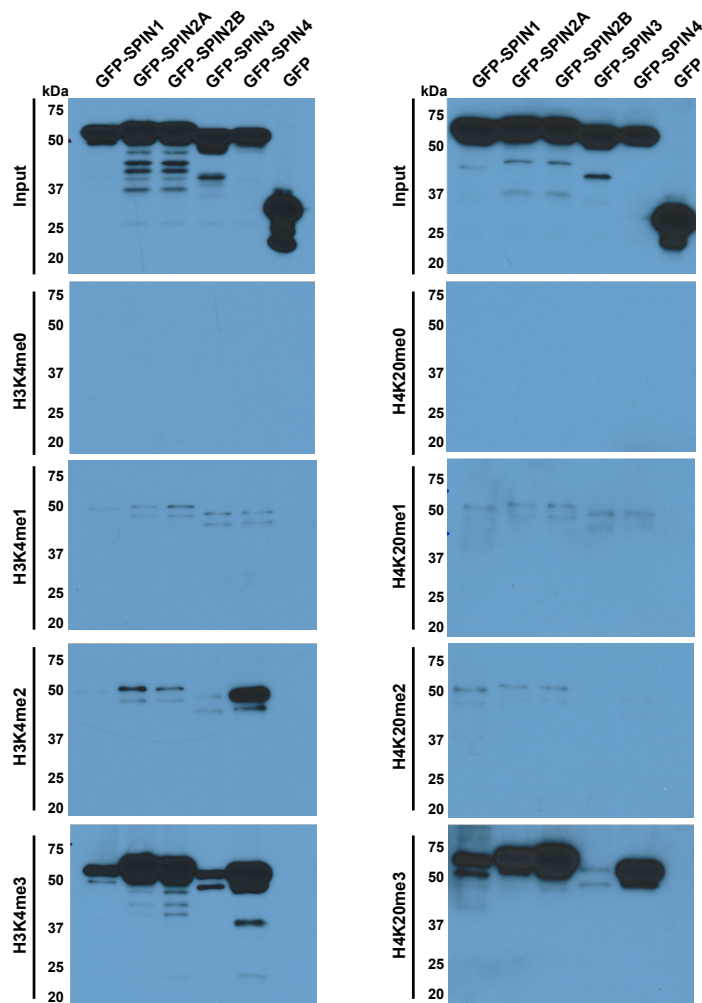

Supplement: Supplementary file 3 — Additional file 3: Figure S1. SPIN1 Tudor domain interaction with H3K4me3 and H4K20me3. A) Representative array images of SPIN1 Tudor domain showing peptide binding indicated in red (right panel). The peptide tracer is shown in green (left panel). Positive antibody controls are outlined in white. B) Western blot results of peptide pull-down experiments with SPIN1 tandem Tudor domain. The input is shown in Lane 1 and the corresponding bound fraction is shown in Lanes 2–8. C) Western blot results of peptide pull-down experiments with whole cell lysates derived from transiently transfected HEK 293T cells (GFP-SPIN1, 2A, 2B, 3 and 4). [file 13072_2017_117_MOESM3_ESM.pdf]

# Figure S2

## A

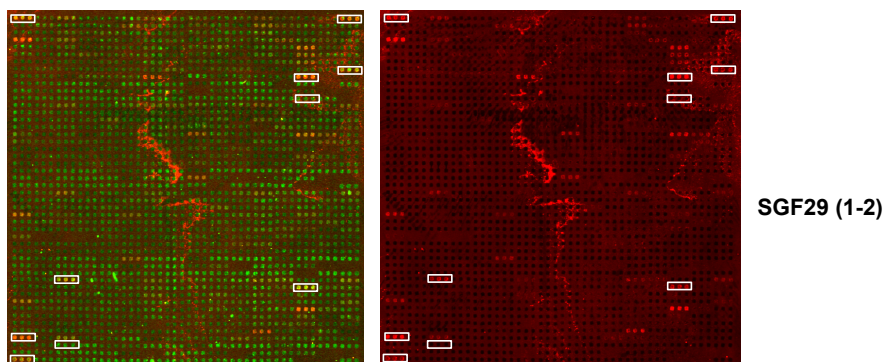

## B

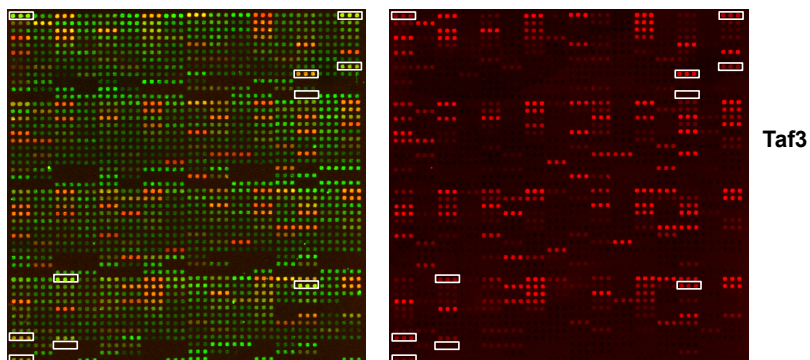

Supplement: Supplementary file 4 — Additional file 4: Figure S2. Peptide microarray data for SGF29 and Taf3. Representative array images of A) SGF29 double Tudor domain and Taf3 PHD domain showing peptide binding indicated in red (right panel). The peptide tracer is shown in green (left panel). Positive antibody controls are outlined in white. [file 13072_2017_117_MOESM4_ESM.pdf]

# Figure S3

## A

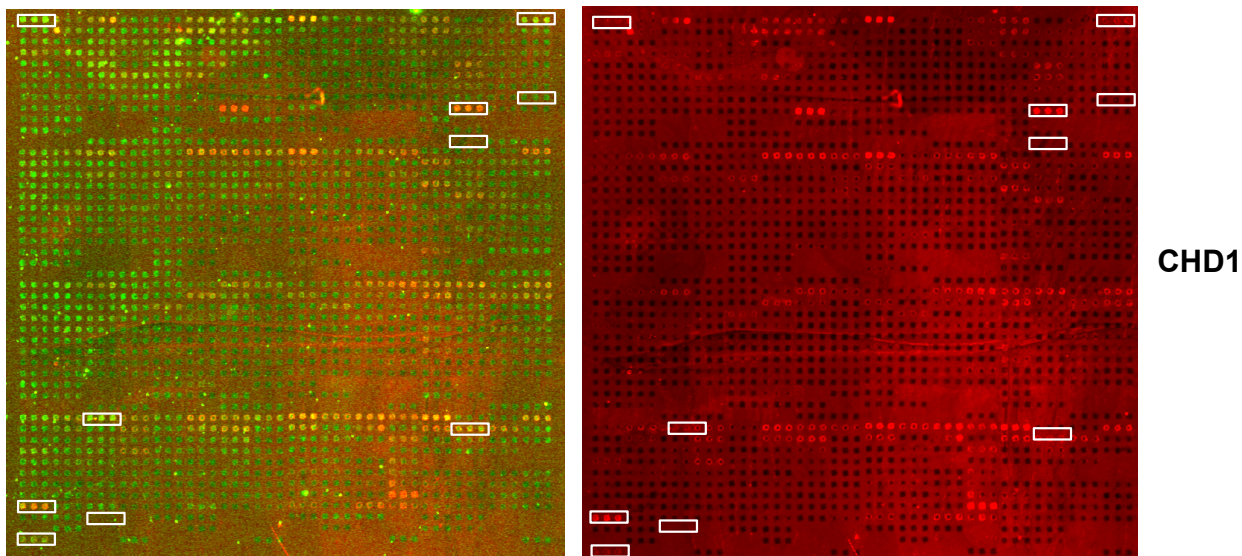

## B

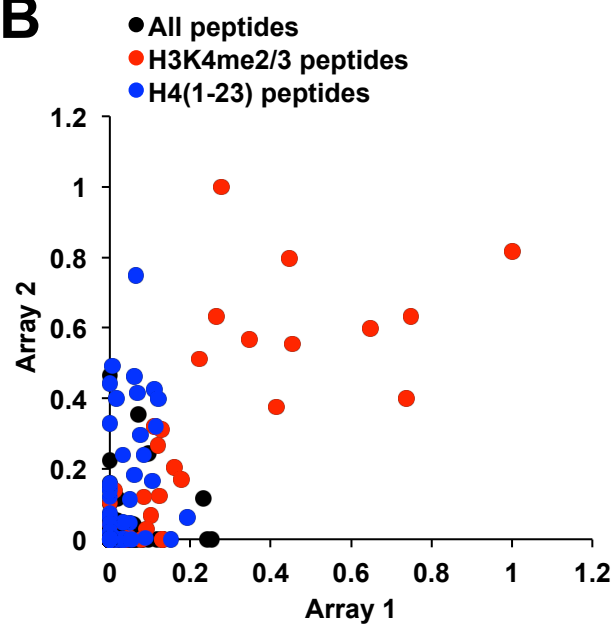

## C

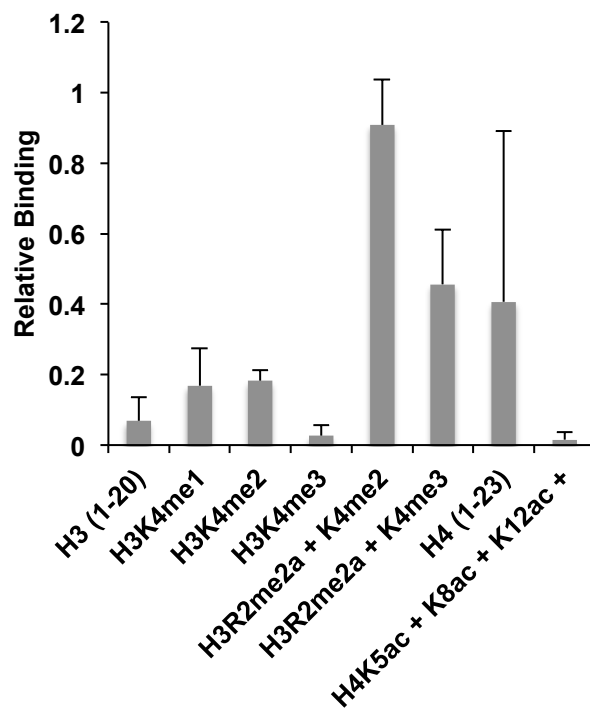

Supplement: Supplementary file 5 — Additional file 5: Figure S3. CHD1 chromodomain histone peptide microarray. A) Representative array images of CHD1 chromodomain showing peptide binding indicated in red (right panel). The peptide tracer is shown in green (left panel). Positive antibody controls are outlined in white. B) Scatter plot of the relative binding of CHD1 chromodomain from two independent peptide arrays. All modified and unmodified H4 (1–23) peptides are shown in blue, and H3K4me2/3-containing peptides are shown in red. All other peptides are shown in black. C) Relative binding to the indicated histone peptides from two representative arrays. Data were normalized to the most intense binding and the average and standard deviation of triplicate spots is shown. [file 13072_2017_117_MOESM5_ESM.pdf]

# Figure S4

## A

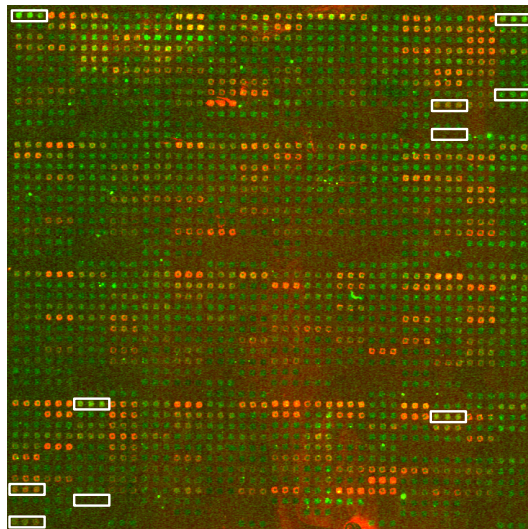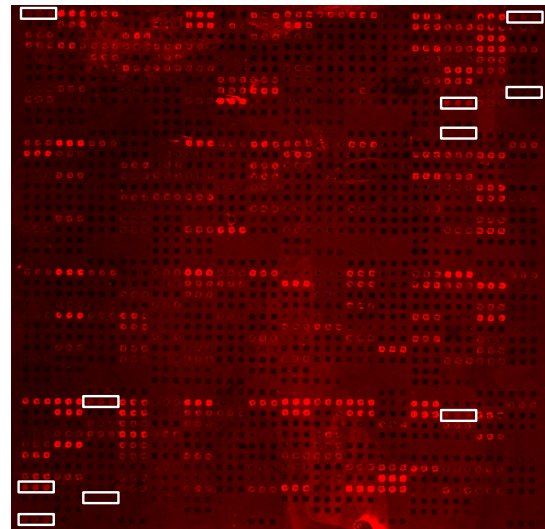

CHD7

## B

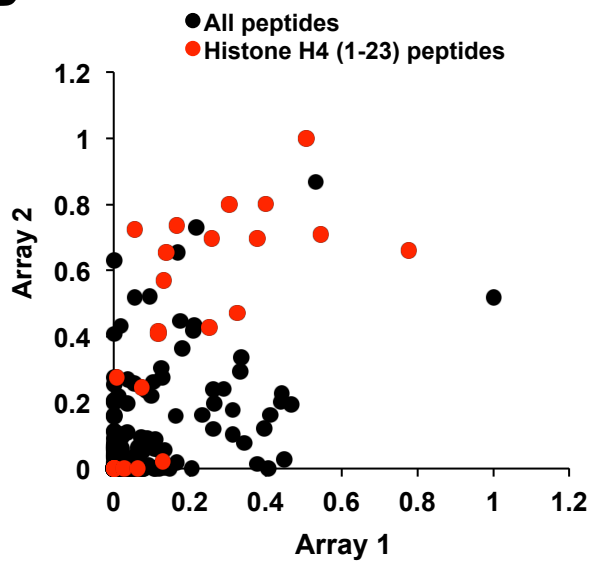

## C

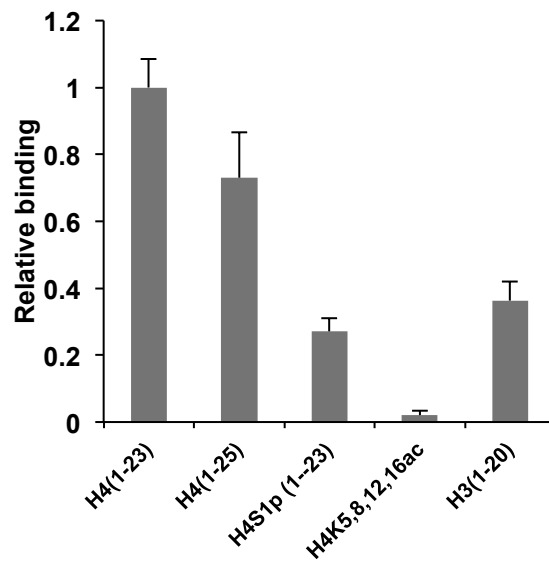

Supplement: Supplementary file 6 — Additional file 6: Figure S4. CHD7 chromodomain histone peptide microarray. A) Representative array images of CHD7 chromodomain showing peptide binding indicated in red (right panel). The peptide tracer is shown in green (left panel). Positive antibody controls are outlined in white. B) Scatter plot of the relative binding of CHD7 chromodomain from two independent peptide arrays. All modified and unmodified H4 (1–23) peptides are shown in red. All other peptides are shown in black. C) Relative binding to the indicated histone peptides from one representative array. Data were normalized to the most intense binding and the average and standard deviation of triplicate spots is shown. [file 13072_2017_117_MOESM6_ESM.pdf]

Figure S5

A

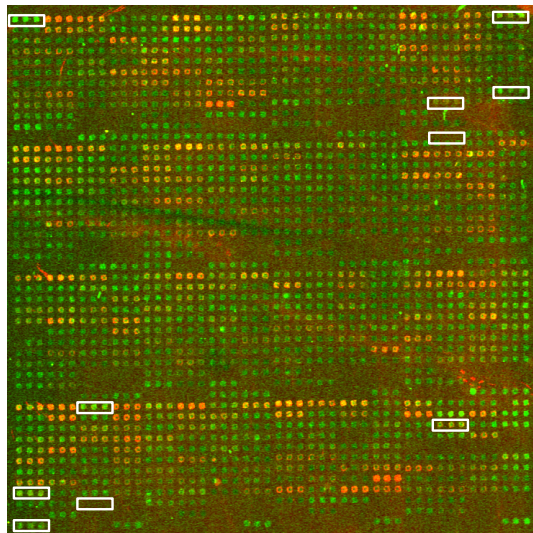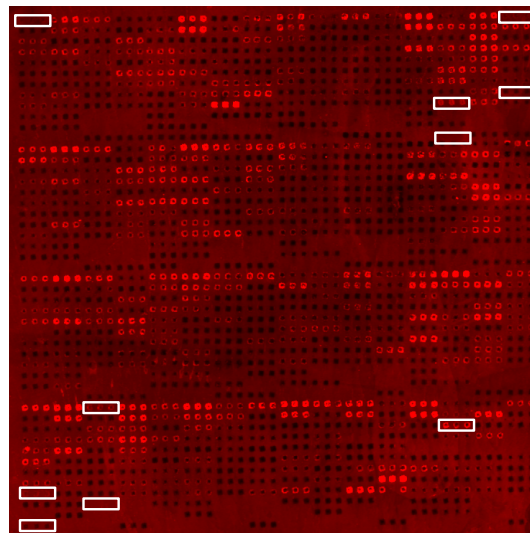

CHD9

B

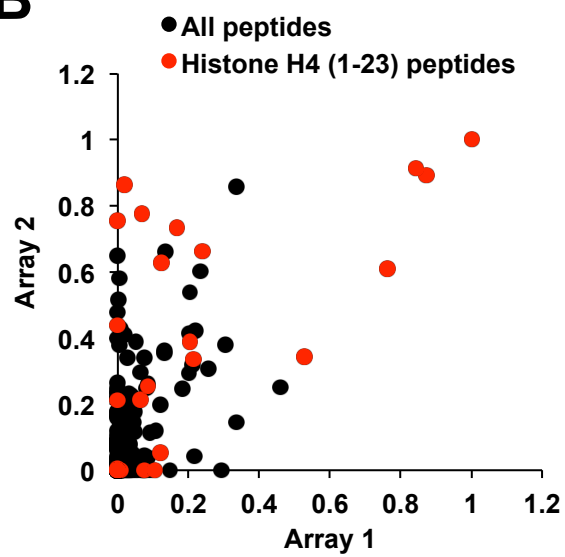

C

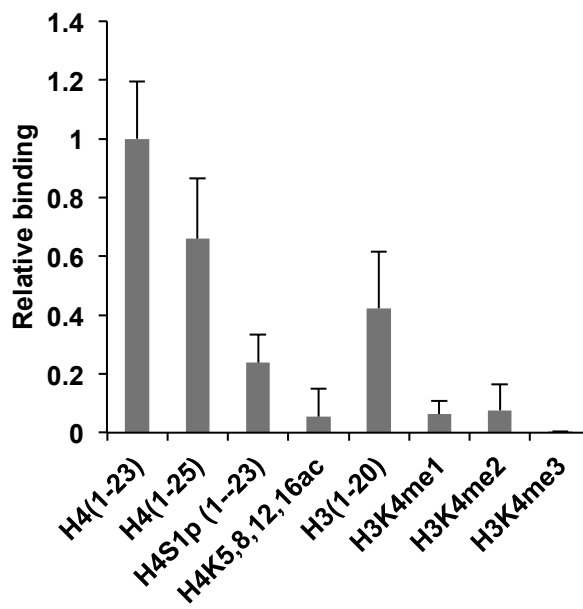

Supplement: Supplementary file 7 — Additional file 7: Figure S5. CHD9 chromodomain histone peptide microarray. A) Representative array images of CHD9 chromodomain showing peptide binding indicated in red (right panel). The peptide tracer is shown in green (left panel). Positive antibody controls are outlined in white. B) Scatter plot of the relative binding of CHD9 chromodomain from two independent peptide arrays. All modified and unmodified H4 (1–23) peptides are shown in red. All other peptides are shown in black. C) Relative binding to the indicated histone peptides from one representative array. Data were normalized to the most intense binding and the average and standard deviation of triplicate spots is shown. [file 13072_2017_117_MOESM7_ESM.pdf]

**Figure S6**

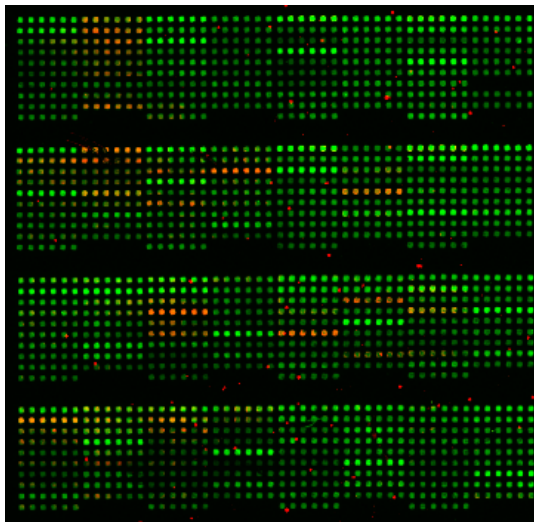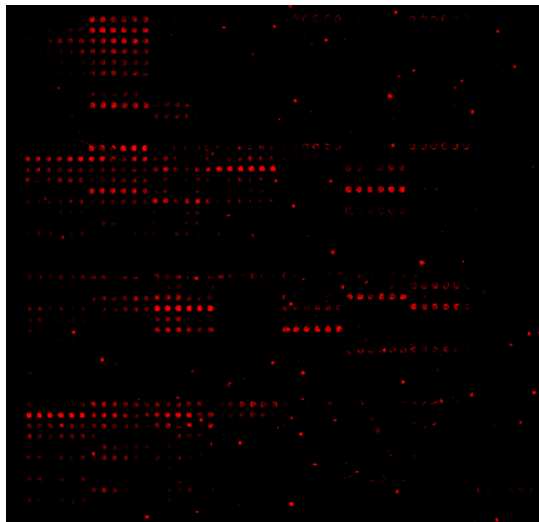

**TDRD3**

Supplement: Supplementary file 8 — Additional file 8: Figure S6. TDRD3 Tudor domain histone peptide microarray. A) Representative array images of TDRD3 Tudor domain showing peptide binding indicated in red (right panel). The peptide tracer is shown in green (left panel). [file 13072_2017_117_MOESM8_ESM.pdf]
